# Combining Natural and Microbial Insecticides with Diatomaceous Earth for Effective Management of *Prostephanus truncatus*

**DOI:** 10.3390/insects16111162

**Published:** 2025-11-14

**Authors:** Demeter Lorentha S. Gidari, Maria C. Boukouvala, Constantin S. Filintas, Anna Skourti, Nickolas G. Kavallieratos

**Affiliations:** Laboratory of Agricultural Zoology and Entomology, Department of Crop Science, Agricultural University of Athens, 75 Iera Odos Str., 11855 Athens, Greece; dlgidari@aua.gr (D.L.S.G.); mbouk@aua.gr (M.C.B.); kfilintas@aua.gr (C.S.F.); annaskourti@aua.gr (A.S.)

**Keywords:** larger grain borer, diatomaceous earth, spinosad, abamectin, *Beauveria bassiana*, stored maize, complete mortality, progeny suppression

## Abstract

*Prostephanus truncatus* is well known for causing extensive postharvest losses and threatening food security in tropical and subtropical regions. The reliance on synthetic insecticides has raised concerns about the development of resistance, environmental safety, food contamination, and human health, emphasizing the need for alternative management strategies. Thus, the present study evaluated the insecticidal efficacy of diatomaceous earth (DE) in the Protector formula alone and in combination with spinosad, abamectin, or the entomopathogenic fungus *Beauveria bassiana* against *P. truncatus* adults and their progeny production on maize under two temperatures (25 and 30 °C) at low concentrations. Bioassays revealed that DE or *B. bassiana* alone induced low mortality, while spinosad and abamectin single applications resulted in high mortality rates, achieving 100% after 21 days of exposure at 30 °C at the higher concentrations. The combined applications of spinosad or abamectin with DE resulted in complete mortality and progeny suppression at lower concentrations at 25 °C. Temperature had a significant influence in some cases. Overall, these findings demonstrate that DE, when combined with other natural insecticidal agents at low concentrations, especially spinosad and abamectin at specific temperatures, can provide highly effective control of *P. truncatus*, offering a promising strategy for integrated pest management (IPM) in stored maize.

## Simple Summary

The larger grain borer, *Prostephanus truncatus*, is a serious pest of stored maize. In this study, diatomaceous earth (DE) was tested against *P. truncatus*, alone and in combination with three other natural or microbial insecticidal agents: spinosad, abamectin, and the entomopathogenic fungus (EPF) *Beauveria bassiana*, at low concentrations, at 25 and 30 °C. The combined applications of DE and EPF resulted in moderate mortality rates and control of the progeny production of *P. truncatus*. On the other hand, spinosad and abamectin combined with DE achieved complete mortality and progeny suppression of *P. truncatus.* Temperature affected the effectiveness of the insecticidal agents significantly in certain cases. These results show that the combination of specific insecticidal agents at low concentrations and selected temperatures can totally control *P. truncatus* on stored maize.

## 1. Introduction

The larger grain borer, *Prostephanus truncatus* (Horn) (Coleoptera: Bostrychidae), is a major pest of stored grains, particularly maize, and dried cassava [1]. Native to Central America, *P. truncatus* became a major intercontinental pest following accidental introductions worldwide and it is now a critical threat to food security, especially in tropical and subtropical regions [2]. In poorly managed, heavily infested conventional storage, *P. truncatus* causes weight losses of up to 40% over three months [3]. Beyond direct weight loss of stored products, *P. truncatus* elevates economic costs by degrading grain quality, e.g., causing broken kernels or contamination with frass and powder, increasing susceptibility to secondary pest infestation, and bacterial and fungal colonization and therefore mycotoxin accumulation [4,5].

Conventional control strategies for *P. truncatus* depend on chemical treatments, including fumigation and surface-applied insecticides, to protect stored grains from infestation [6]. For the last three decades, pyrethroids and organophosphates have been the major group of insecticides used in control measures against *P. truncatus* worldwide [7,8]. Successful suppression of *P. truncatus* infestations in stored maize has been achieved using pyrethroid insecticides with active ingredients such as deltamethrin, permethrin, and alpha-cypermethrin as grain protectants [1,9].

The widespread application of chemical insecticides has led to the development of resistance among pest populations, environmental concerns, and instances of human poisoning [10,11,12]. These challenges associated with chemical control of stored-product pests have driven research into alternative management strategies. Among them, entomopathogenic fungi (EPF), diatomaceous earth (DE), and naturally derived products, such as spinosad, have gained attention as more sustainable and environmentally friendly options [13,14,15]. DEs consist of fossilized diatom shells rich in amorphous silica, which abrade the insect cuticle, causing desiccation and mortality, and are commercially available for controlling a wide range of stored-product pests [16]. DEs have reported insecticidal efficacy against major stored maize pests, such as *P. truncatus*, *Sitophilus zeamais* (Motschulsky) (Coleoptera: Curculionidae), and *Tribolium castaneum* (Herbst) (Coleoptera: Tenebrionidae) [17]. It has been previously demonstrated that effective control of insect pests with DEs require application rates of at least 1000–3500 ppm [14,18]. However, such high concentrations raise concerns regarding worker safety and grain quality, including reductions in bulk density [14,19].

To increase the effectiveness and keep the application concentrations of DEs low, researchers have suggested their combination with other control agents of natural origin, such as botanicals [20], microbe metabolites [17], synthetic compounds [21], and insect growth regulators [22]. Spinosad, derived from *Saccharopolyspora spinosa* Mertz and Yao (Bacteria: Pseudonocardiaceae), targets the insect nervous system, causing mortality, while exhibiting low toxicity to mammals, birds, and beneficial organisms [23]. EPFs, including *Beauveria bassiana* (Balsamo) Vuillemin (Hypocreales: Cordycipitaceae), infect their hosts by attaching to and penetrating the insect cuticle, proliferating internally, producing toxins, and ultimately causing host death [24]. Various DEs, EPFs, and spinosad are already commercially available as natural control agents with strong insecticidal properties against a wide range of stored-product pests [25,26,27,28]. Avermectins, such as abamectin, are extensively applied in veterinary medicine as insecticides and acaricides due to their broad-spectrum activity against ectoparasites like mites, lice, and ticks [29]. They act on glutamate-gated chloride channels in invertebrates, causing paralysis and death, while exhibiting low mammalian toxicity when used at recommended concentrations [30,31]. These compounds are administered to livestock in pour-on, injectable, or oral formulations, providing protection against endo- and ectoparasites in horses, pigs, sheep, and goats [32].

In recent years, the use of DEs in combination with other insecticides has gained considerable scientific attention, achieving effective control of stored-product insects, even at low application concentrations [33,34]. For instance, Gad et al. [35] demonstrated that combining DEs with spinosad or the EPF *Trichoderma harzianum* Rifai (Sordariomycetes: Hypocreaceae) significantly increased mortality rates vs. single applications in *Sitophilus oryzae* (L.) (Coleoptera: Curculionidae), leading to the death of all exposed individuals within 7 days of exposure.

Although both single and combined applications of DEs with other control agents, such as EPFs and spinosad, have been previously studied and shown to produce additive effects, the DE formulation Protector has not yet been evaluated alone or in combination with EPFs, spinosad, or abamectin for the management of *P. truncatus*. To address this gap, the present study aimed to evaluate the insecticidal efficacy of three combinations—DE + spinosad, DE + *B. bassiana*, and DE + abamectin—against adults of *P. truncatus*, as well as their effects on progeny production.

## 2. Materials and Methods

### 2.1. Prostephanus truncatus Rearing and Grain Substrate

Adults of *P. truncatus* were maintained on whole maize kernels under constant darkness at 30 °C and 65% relative humidity (RH) in the Agricultural Zoology and Entomology Laboratory at the Agricultural University of Athens. For the bioassays, unsexed adults less than two weeks old were selected [36]. Clean maize, *Zea mays* L. (var. Dias), free from prior infestation or pesticide residues, was used in the trials. Prior to experimentation, maize moisture was recorded at 11.8% using a moisture meter (Holzfeuchte Messgerät/1, Einhell SAS, Villepinte, France).

### 2.2. Insecticidal Agents

Four insecticidal agents were used in the trials: (1) Protector, a DE formulation containing 66.8% SiO_2_ and 10% silica gel [37] (Intrachem Bio Italia, Grassobbio, Bergamo, Italy); (2) Laser SC, with 48% spinosad as its active ingredient (a.i.) (Dow Agrosciences Export S.A.S., Lavrion, Greece); (3) Vertimec EC, an emulsifiable concentrate formulation containing 1.8% w/v abamectin (a.i.) (Syngenta, Attika, Greece); and (4) the fungal insecticide Naturalis OD (oil dispersion), containing *B. bassiana* (strain ATCC 74040) at 2.3 × 10^7^ conidia/mL (CBC (Europe), Grassobbio, Italy). The two tested dilutions (2.3 × 10^4^ and 2.3 × 10^6^ conidia/mL) were prepared in distilled water.

### 2.3. Bioassays

Three concentrations of spinosad (0.01, 0.1, and 0.5 ppm) [38] and one concentration of the DE (150 ppm) [39] were evaluated prior to the trials. Eight lots (1 kg each) of maize were used in the trials. One of the lots was used as an untreated control. For each concentration of spinosad (0.01, 0.1, and 0.5 ppm), one lot was sprayed with 1 mL of the respective insecticidal concentration, using an airbrush (Everything Airbrush, Poole, UK). The treated maize lots were placed into different glass jars of 5 L and shaken for 10 min [40], manually, until the uniform spread of the insecticide in the maize mass was achieved. This procedure was performed once again, and the spinosad-treated lots were then mixed, after they had dried, with 150 ppm of the DE and manually shaken for 10 min to achieve an equal distribution of DE particles in the maize mass. One lot was treated with 150 ppm of the DE, as described above, and the remaining maize lot was kept untreated to be used as the control. Then, three 20 g samples of each treated and untreated lot were weighed on a layer using a compact balance (Adam Equipment Co Ltd., Milton Keynes, UK) and transferred, using different scoops for each jar, into separate glass vials (7 cm × 10 cm diameter × height). To prevent individuals from escaping, the internal upper part of the vials was coated with polytetrafluoroethylene (Nanographi, Ankara, Turkey). Twenty *P. truncatus* adults were placed into each vial and transferred to an incubator set at 25 °C, 65% RH and constant darkness. All vials were gauze-covered for aeration [40]. Adult mortality was assessed after 7, 14, and 21 days, using an Olympus stereomicroscope (Olympus SZX9; Bacacos S.A., Athens, Greece) under 40× total magnification. All *P. truncatus* adults (dead and alive) were removed and discarded from the vials after the 21 days. Next, the vials were placed into the incubator, under the same conditions, for an additional 46 days [36]. After this period, the progeny production was estimated by counting the emerged *P. truncatus* adults. The hole experimentation was performed three times. Trials corresponding to abamectin (0.001, 0.01, and 0.1 ppm) [41], *B. bassiana* (2.3 × 10^4^ and 2.3 × 10^6^ conidia/mL) (based on preliminary bioassays), and their respective combinations with the DE were conducted as described above, using different lots of maize, vials, individuals, and airbrushes. All the trials were also performed at 30 °C.

### 2.4. Statistical Analysis

Since the mortalities were below 5% in the controls, no adjustments were made to the data analysis for mortality in this study. The dataset was transformed into log(x + 1), prior to the analysis, to standardize variance [42]. Mortality data of each tested insecticidal agent or combination underwent analysis using the model of repeated measures [43], with treatment and temperature as the main effects, exposure intervals as the repeated factors, and mortality as the response variable. For the progeny data, a two-way ANOVA [43] was conducted with temperature and treatment as main effects. Number of offspring was the response variable. Using the Tukey HSD test or the two-tailed *t*-test with n − 2 df at a significance level of 0.05 [44], means were separated. Each analysis was performed using the statistical package JMP 16.2 [45].

## 3. Results

### 3.1. Prostephanus truncatus Adult Mortality

#### 3.1.1. Spinosad + DE Trials

For *P. truncatus* adults treated with spinosad, DE, or their combinations, between exposures, the main effects, along with the associated interaction were significant. Within exposures, only exposure and exposure × treatment were significant (Table 1). Concerning the 7-day mortality count, the DE alone resulted, at both temperatures (25 and 30 °C), in significantly lower mortality rates (7.2 and 11.7%, respectively) compared to all the other treatments. The highest mortality rates were observed with the combination of 0.5 ppm of spinosad + 150 ppm of the DE (92.2 and 95.0%, at 25 and 30 °C, respectively), and they did not significantly differ from those of all the other combined treatments or the 0.1 or 0.5 ppm spinosad single applications. After 14 and 21 days, the single application of the DE resulted in significantly lower mortality rates (19.2–46.4% at 25 °C and 26.9–66.1% at 30 °C) compared to all the other single or combined applications. The combination of 0.5 ppm of spinosad + 150 ppm of the DE achieved 100% mortality of *P. truncatus* adults 14 days post-exposure at 30 °C. Complete mortality was observed at the 21-day count for the combination of 0.5 ppm of spinosad + 150 ppm of the DE and spinosad 0.5 ppm at 25 °C and all the combined applications at 30 °C. After 21 days of exposure, the mortality rates of *P. truncatus* adults exposed to 150 ppm of the DE and to the combination of 0.01 ppm of spinosad + 150 ppm of the DE significantly differed between the two temperatures (25 and 30 °C), with higher mortality rates at 30 °C (Table 2).

#### 3.1.2. *Beauveria bassiana* + DE Trials

Concerning the *P. truncatus* adults exposed to *B. bassiana*, DE, or their combinations, between and within exposures, temperature, treatment, exposure, and exposure × treatment were significant (Table 3). Seven days post-exposure, there were no significant differences among the treatments. However, the single application of *B. bassiana* 2.3 × 10^6^ conidia/mL and the combination of *B. bassiana* 2.3 × 10^6^ conidia/mL + 150 ppm of the DE mortality rates significantly differed between the two temperatures (25 and 30 °C) (7.5 vs. 10.8% and 9.4 vs. 12.8%, respectively). After 14 days of exposure, at 30 °C, the mortality rates of the single application of the DE and the combination with *B. bassiana* 2.3 × 10^6^ conidia/mL were significantly higher (26.9 and 28.3%, respectively) compared to *B. bassiana* 2.3 × 10^4^ conidia/mL single application and the *B. bassiana* 2.3 × 10^4^ conidia/mL + DE 150 ppm combination (18.6 and 20.3%, respectively). Concerning the two temperatures, the same pattern as for the 7-day interval was observed with the addition of the single application of the DE. At the 21-day mortality count, the single application of the DE and both the combined treatments resulted in significantly higher mortality rates compared to the *B. bassiana* single applications. Except for *B. bassiana* 2.3 × 10^4^ conidia/mL, all the other insecticidal applications significantly differed between the two temperatures (25 and 30 °C), with higher mortality rates at 30 °C. In all treatments, mortality did not exceed 70.3% (Table 4).

#### 3.1.3. Abamectin + DE Trials

Temperature, treatment, exposure, and exposure × treatment were significant (Table 5). The 7-day mortality count revealed that, at 25 °C, the rates of single applications of the DE (8.9%) and abamectin 0.001 ppm (15.6%) significantly differed, while they were significantly lower compared to the other treatments. At 30 °C, the applications of abamectin 0.1 ppm and the combinations of the DE with abamectin 0.01 or 0.1 ppm led to higher mortality rates (85.3, 82.2, and 91.4%, respectively) compared to the other treatments. The single application of the DE and the combination with abamectin 0.01 ppm revealed significant differences between the two temperatures (25 and 30 °C), with higher mortality rates at 30 °C. Fourteen days post-exposure, at both temperatures (25 and 30 °C), the single application of abamectin 0.1 ppm and the combinations of 0.01 or 0.1 ppm + the DE resulted in significantly higher mortality rates vs. the other treatments. The exposure to abamectin 0.1 ppm or abamectin 0.01 + DE revealed significant differences between the two temperatures (25 and 30 °C), with mortality rates at 30 °C exceeding those at 25 °C. Concerning the 21-day mortality count, complete mortality was observed with abamectin 0.1 (30 °C), abamectin 0.01 + DE (30 °C), and abamectin 0.1 + DE (25 and 30 °C). The single applications of abamectin 0.1 ppm or DE 150 ppm led to significantly higher mortalities at 30 °C compared to 25 °C (Table 6).

### 3.2. Prostephanus truncatus Progeny Production

#### 3.2.1. Spinosad + DE Trials

Treatment and temperature × treatment were significant, while temperature was not (Table 7). After the exposure to 0.01, 0.1, and 0.5 ppm of spinosad, 150 ppm of the DE, or their combinations at 25 and 30 °C, the two higher concentrations of spinosad (0.1 and 0.5 ppm), along with all the combined treatments, achieved progeny suppression, significantly differing from the control and the single DE application. The control and the single application of the DE revealed significantly higher progeny production at 30 °C compared to 25 °C (38.6 vs. 26.8 and 23.0 vs. 12.4 adults/vial, respectively) (Table 8).

#### 3.2.2. *Beauveria bassiana* + DE Trials

Both main effects (temperature and treatment) along with their associated interaction were significant (Table 9). None of the treatments were able to suppress *P. truncatus* progeny production; however, all the applications at both temperatures resulted in significantly lower progeny production of *P. truncatus* (4.3–22.6 adults/vial) compared to the control (26.3 and 37.2 adults/vial, at 25 and 30 °C, respectively). For the control, *B. bassiana* 2.3 × 10^6^ conidia/mL single application, DE single application, and *B. bassiana* 2.3 × 10^4^ conidia/mL ppm + DE, progeny production significantly differed between 25 and 30 °C (Table 10).

#### 3.2.3. Abamectin + DE Trials

The main effect of treatment and the interaction of temperature × treatment were significant (Table 11). At both temperatures (25 and 30 °C), the higher concentrations of abamectin (0.01 and 0.1 ppm) as well as all the combined treatments achieved progeny suppression, significantly differing from the control, the lower concentration of abamectin (0.001 ppm), and the DE single application. Control, abamectin 0.001 ppm, and the DE treatments significantly differed between 25 and 30 °C (26.9 vs. 36.8, 2.8 vs. 5.9, and 11.7 vs. 21.8 adults/vial, respectively) (Table 12).

## 4. Discussion

The combined applications of the DE with spinosad, *B. bassiana*, and abamectin, as maize protectants against *P. truncatus*, were evaluated and found to result in higher mortality rates and lower progeny production compared to the single applications. However, the recorded efficacy varied among treatments. The spinosad + DE and the abamectin + DE treatments achieved complete *P. truncatus* adult mortality and progeny suppression, while the *B. bassiana* + DE combination led to moderate results.

Spinosad in single applications was very effective against *P. truncatus* adults, achieving mortality rates > 90% after 7 days of exposure or progeny suppression, agreeing with previous research [1,38,46]. The combined applications with the DE led to further efficacy (complete mortality), although differences among combined and single spinosad treatments were not significant in most cases. A similar pattern was also observed for the abamectin single and DE combined applications. The fact that the DE enhanced the action of both insecticides, but not significantly, is possibly due to the modes of action of the three insecticidal agents. Spinosad targets the gamma aminobutyric acid and nicotinic receptors of the nervous system [47,48]. Abamectin acts on gamma aminobutyric acid receptors in a different way to spinosad [49,50], causing paralysis in insects, leading to death [51]. Both spinosad and abamectin act with lethal consequences for insects in a short period of time [17,41,46,52,53,54,55]. On the other hand, DEs are slow-acting substances [26]. The protective wax layer on the cuticle of insects is harmed by the deposited particles of the DEs, primarily through sorption and to a lesser extent through abrasion, or both. The primary factor that causes insects to die is the loss of water from their bodies due to desiccation [14]. Ebeling [56] stated that insects die after losing ~60% of their water content; therefore, insect mortality takes time to occur. Given that spinosad and abamectin act fast, while the DE is slow-acting, their combinations did not result in significantly improved efficiency against *P. truncatus*, as spinosad and abamectin kill individuals before the DE starts killing. Additionally, DE particle retention depends on the surface of each grain, being more efficient at rough surfaces [14]. Considering that maize kernels have a slick surface [57], DE particles probably were not retained on the treated maize, and, as a result, the DE was not effective against *P. truncatus* adults, neither in single applications, nor in combinations with other insecticidal agents.

Among all single applications, *B. bassiana*, at both concentrations, resulted in the lowest mortality rates of *P. truncatus* adults. *Beauveria bassiana* single applications did not achieve the suppression of *P. truncatus* progeny production. EPF single treatments do not show high insecticidal efficacy against stored-product pests. However, the combination of *B. bassiana* with other insecticidal agents improves their effectiveness [40,58,59,60]. The insecticidal efficacy of an EPF depends on multiple factors, such as the environmental conditions, the species or strain of the EPF, the targeted insect species, and the type of grain [27,60,61,62,63]. A high level of RH (93–96.5%) is a crucial environmental parameter for the fungi [64]. Given that storage environments have notably lower RH levels, creating unfavorable environments for some fungal strains, this may explain the low insecticidal efficacy [64,65]. Additionally, different strains/isolates of the same EPF may result in different insecticidal efficacies. Specifically for *P. truncatus*, multiple isolates of *B. bassiana* resulted in significantly different mortality rates, with some of them achieving complete mortality 4 days post-exposure, while others did not exceed 85% [66]. Using a different isolate, Nboyine et al. [67] managed to achieve up to 50% mortality of *P. truncatus* adults. In the current study, the combination of *B. bassiana* with the DE improved the effectiveness of the EPF against *P. truncatus*, especially the mortality rates, at both 25 and 30 °C, 21 days post-exposure, where the combined applications significantly differed from the single fungal treatment. This improvement may be due to the increased adhesion level of the *B. bassiana* conidia to the cuticle of *P. truncatus* adults through the presence of the DE particles [25].

The results of the current study indicated that temperature was a critical factor modulating, significantly in many cases, the efficacy of the tested agents and their combinations. It plays a crucial role in the action of the insecticidal agents [40,60,68]. *Beauveria bassiana* shows the fastest germination rate at 25–32 °C, while the fastest growth is observed at 30 °C [69]. High temperatures increase dehydration in adult individuals under exposure to DEs, since they absorb and damage the insect cuticular wax, thus leading to elevated mortality levels [14,70,71,72]. In general, the influence of temperature on the metabolic activities of insects affects the action of an insecticide. High temperatures increase those activities, further stressing the insect under the influence of the insecticidal agent [46].

## 5. Conclusions

The findings of the present study highlight the potential of combining insecticidal agents with DE as an effective management strategy for *P. truncatus* in stored maize. Notably, the combinations of spinosad or abamectin with DE achieved complete mortality of *P. truncatus* adults and suppressed progeny production at all concentrations. From the IPM point of view, these results suggest that the quantities of natural/microbial insecticides can be reduced and that, combined with DEs, they can achieve the highest efficiency. However, future studies are needed to test these combinations against other stored-product pests, check the efficacy of different species or strains of EPF, and evaluate the influence of other biotic factors, such as RH.

## Figures and Tables

**Table 1:** MANOVA parameters depicting the main effects and their interactions leading to the observed mortalities of *Prostephanus truncatus* adults treated with spinosad (0.01, 0.1, and 0.5 ppm), DE (150 ppm), or their combinations, between and within exposures (error df = 154).

Between Exposure Intervals			
Source	df	*F*	*p*
Intercept	1	75,926.3	<0.01
Temperature	1	12.8	<0.01
Treatment	6	186.8	<0.01
Temperature × treatment	6	5.8	<0.01
** Within Exposure Intervals **			
** Source **	** df **	*** F ***	*** p ***
Exposure	2	166.5	<0.01
Exposure × temperature	2	1.8	0.17
Exposure × treatment	12	33.7	<0.01
Exposure × temperature × treatment	12	0.5	0.90

**Table 2:** Mortality (%, mean ± SE) of *Prostephanus truncatus* adults after 7, 14, and 21 days in maize treated with spinosad (0.01, 0.1, and 0.5 ppm), DE (150 ppm), or their combinations at 25 and 30 °C. Within each row, asterisks indicate significant differences (df = 22; two-tailed *t*-test at *p* = 0.05). Within each temperature, per exposure, means followed by the same uppercase letter are not significantly different (df = 6, 83; Tukey HSD test at *p* = 0.05). For entries marked with dashes, no analysis was conducted.

	Temperature	25 °C	30 °C	*t*	*p*
Treatment					
7 days					
Spinosad 0.01		45.0 ± 3.9 B	54.7 ± 5.1 B	1.3	0.21
Spinosad 0.1		77.5 ± 3.7 A	84.7 ± 1.8 A	1.9	0.08
Spinosad 0.5		90.8 ± 1.8 A	92.5 ± 2.1 A	0.6	0.57
DE		7.2 ± 1.6 C	11.7 ± 1.7 C	2.0	0.06
Spinosad 0.01 + DE		73.1 ± 3.1 AB	76.9 ± 2.7 A	1.0	0.34
Spinosad 0.1 + DE		82.5 ± 2.9 A	89.4 ± 2.2 A	2.0	0.06
Spinosad 0.5 + DE		92.2 ± 2.7 A	95.0 ± 0.9 A	1.1	0.29
*F*		62.8	117.7		
*p*		<0.01	<0.01		
14 days					
Spinosad 0.01		87.5 ± 2.3 A	90.6 ± 2.0 A	1.0	0.33
Spinosad 0.1		93.6 ± 2.2 A	95.6 ± 1.2 A	0.8	0.42
Spinosad 0.5		96.4 ± 1.3 A	97.2 ± 0.8 A	0.6	0.58
DE		19.2 ± 3.2 B	26.9 ± 1.8 B	2.0	0.06
Spinosad 0.01 + DE		92.8 ± 1.6 A	91.4 ± 3.0 A	−0.5	0.62
Spinosad 0.1 + DE		96.4 ± 1.5 A	99.2 ± 0.4 A	1.8	0.09
Spinosad 0.5 + DE		98.6 ± 0.8 A	100.0 ± 0.0 A	1.8	0.08
*F*		36.8	285.3		
*p*		<0.01	<0.01		
21 days					
Spinosad 0.01		93.3 ± 2.1 A	96.7 ± 1.5 A	1.3	0.22
Spinosad 0.1		96.1 ± 1.3 A	98.3 ± 0.9 A	1.4	0.17
Spinosad 0.5		99.2 ± 0.4 A	100.0 ± 0.0 A	1.9	0.07
DE		46.4 ± 2.1 B	66.1 ± 2.3 B *	6.1	<0.01
Spinosad 0.01 + DE		96.4 ± 1.4 A	100.0 ± 0.0 A *	2.5	0.02
Spinosad 0.1 + DE		98.9 ± 0.7 A	100.0 ± 0.0 A	1.5	0.15
Spinosad 0.5 + DE		100.0 ± 0.0 A	100.0 ± 0.0 A	-	-
*F*		175.7	106.5		
*p*		<0.01	<0.01		

**Table 3:** MANOVA parameters depicting the main effects and their interactions leading to the observed mortalities of *Prostephanus truncatus* adults treated with *Beauveria bassiana* (2.3 × 10^4^ and 2.3 × 10^6^ conidia/mL), DE (150 ppm), or their combinations, between and within exposures (error df = 154).

Between Exposure Intervals			
Source	df	* F *	* p *
Intercept	1	13,776.7	<0.01
Temperature	1	33.7	<0.01
Treatment	4	12.2	<0.01
Temperature × treatment	4	0.8	0.52
** Within Exposure Intervals **			
** Source **	** df **	*** F ***	*** p ***
Exposure	2	786.3	<0.01
Exposure × temperature	2	1.9	0.15
Exposure × treatment	8	11.6	<0.01
Exposure × temperature × treatment	8	0.7	0.70

**Table 4:** Mortality (%, mean ± SE) of *Prostephanus truncatus* adults after 7, 14, and 21 days in maize treated with *Beauveria bassiana* (2.3 × 10^4^ and 2.3 × 10^6^ conidia/mL), DE (150 ppm), or their combinations at 25 and 30 °C. Within each row, asterisks indicate significant differences (df = 22; two-tailed *t*-test at *p* = 0.05). Within each temperature, per exposure, means followed by the same uppercase letter are not significantly different (df = 4, 59; Tukey HSD test at *p* = 0.05). No significant differences were recorded for entries with no letters.

	Temperature	25 °C	30 °C	*t*	*p*
Treatment					
7 days					
*B. bassiana* 2.3 × 10^4^		6.4 ± 1.0	9.2 ± 1.0	1.7	0.10
*B. bassiana* 2.3 × 10^6^		7.5 ±1.1	10.8 ± 0.8 *	2.1	0.05
DE		7.5 ±1.1	11.9 ± 1.5	1.9	0.07
*B. bassiana* 2.3 × 10^4^ + DE		8.1 ±0.6	11.4 ± 1.3	1.7	0.10
*B. bassiana* 2.3 × 10^6^ + DE		9.4 ± 1.0	12.8 ± 1.0 *	2.4	0.03
*F*		1.2	1.2		
*p*		0.32	0.33		
14 days					
*B. bassiana* 2.3 × 10^4^		15.6 ± 1.3	18.6 ± 1.1 B	1.8	0.09
*B. bassiana* 2.3 × 10^6^		17.8 ± 2.0	24.2 ± 1.5 AB *	2.7	0.01
DE		19.7 ± 2.7	26.9 ± 1.6 A *	2.3	0.03
*B. bassiana* 2.3 × 10^4^ + DE		18.3 ± 0.9	20.3 ± 2.1 B	0.4	0.69
*B. bassiana* 2.3 × 10^6^ + DE		21.4 ± 1.3	28.3 ± 2.0 A *	2.9	0.01
*F*		1.3	5.8		
*p*		0.28	0.01		
21 days					
*B. bassiana* 2.3 × 10^4^		26.4 ± 1.4 B	30.3 ± 2.0 C	1.6	0.13
*B. bassiana* 2.3 × 10^6^		31.9 ± 2.9 B	41.1 ± 2.5 B *	2.5	0.02
DE		46.4 ± 2.3 A	65.8 ± 2.2 A *	5.8	<0.01
*B. bassiana* 2.3 × 10^4^ + DE		53.6 ± 2.0 A	68.1 ± 1.5 A *	5.7	<0.01
*B. bassiana* 2.3 × 10^6^ + DE		57.5 ± 2.5 A	70.3 ± 2.5 A *	3.8	0.01
*F*		35.1	71.0		
*p*		<0.01	<0.01		

**Table 5:** MANOVA parameters depicting the main effects and their interactions leading to the observed mortalities of *Prostephanus truncatus* adults treated with abamectin (0.001, 0.01, and 0.1 ppm), DE (110 ppm), or their combinations, between and within exposures (error df = 154).

Between Exposure Intervals			
Source	df	* F *	* p *
Intercept	1	56,528.4	<0.01
Temperature	1	12.8	<0.01
Treatment	6	149.4	<0.01
Temperature × treatment	6	1.2	0.32
** Within Exposure Intervals **			
** Source **	** df **	*** F ***	*** p ***
Exposure	2	496.4	<0.01
Exposure × temperature	2	1.1	0.33
Exposure × treatment	12	36.0	<0.01
Exposure × temperature × treatment	12	0.45	0.94

**Table 6:** Mortality (%, mean ± SE) of *Prostephanus truncatus* adults after 7, 14, and 21 days in maize treated with abamectin (0.001, 0.01, and 0.1 ppm), DE (150 ppm), or their combinations at 25 and 30 °C. Within each row, asterisks indicate significant differences (df = 22; two-tailed *t*-test at *p* = 0.05). Within each temperature, per exposure, means followed by the same uppercase letter are not significantly different (df = 6, 83; Tukey HSD test at *p* = 0.05). For entries marked with dashes, no analysis was conducted.

	Temperature	25 °C	30 °C	*t*	*p*
Treatment					
7 days					
Abamectin 0.001		15.6 ± 2.9 C	22.2 ± 4.0 C	0.9	0.38
Abamectin 0.01		24.7 ± 2.5 B	30.0 ± 2.1 B	1.8	0.09
Abamectin 0.1		83.3 ± 1.1 A	85.3 ± 1.3 A	1.2	0.26
DE		8.9 ± 0.7 D	12.5 ± 1.3 C *	2.1	0.04
Abamectin 0.001 + DE		26.9 ± 1.8 B	35.0 ± 3.7 B	1.6	0.13
Abamectin 0.01 + DE		76.1 ± 1.8 A	82.2 ± 1.2 A *	2.9	0.01
Abamectin 0.1 + DE		87.8 ± 1.8 A	91.4 ± 1.0 A	1.8	0.08
*F*		119.7	54.9		
*p*		<0.01	<0.01		
14 days					
Abamectin 0.001		39.2 ± 4.5 B	42.2 ± 5.3 BC	0.2	0.86
Abamectin 0.01		45.3 ± 2.4 B	54.7 ± 4.8 B	1.7	0.11
Abamectin 0.1		92.2 ± 1.1 A	96.1 ± 1.0 A *	2.6	0.02
DE		22.8 ± 2.7 C	28.3 ± 1.6 C	1.8	0.08
Abamectin 0.001 + DE		41.1 ± 2.2 B	51.9 ± 5.9 B	1.3	0.20
Abamectin 0.01 + DE		87.8 ± 2.2 A	94.4 ± 1.4 A *	2.6	0.02
Abamectin 0.1 + DE		95.8 ± 1.9 A	97.8 ± 0.7 A	1.0	0.33
*F*		54.4	36.2		
*p*		<0.01	<0.01		
21 days					
Abamectin 0.001		55.6 ± 3.6 CD	58.3 ± 2.9 D	0.7	0.49
Abamectin 0.01		68.1 ± 4.3 BC	73.9 ± 3.7 BC	1.1	0.29
Abamectin 0.1		95.6 ± 1.7 A	100.0 ± 0.0 A *	2.6	0.02
DE		49.4 ± 2.5 D	67.2 ± 2.4 C *	5.0	<0.01
Abamectin 0.001 + DE		70.8 ± 4.5 B	77.8 ± 2.7 B	1.4	0.17
Abamectin 0.01 + DE		99.2 ± 0.6 A	100.0 ± 0.0 A	1.4	0.18
Abamectin 0.1 + DE		100.0 ± 0.0 A	100.0 ± 0.0 A	-	-
*F*		36.1	46.0		
*p*		<0.01	<0.01		

**Table 7:** ANOVA parameters for main effects and associated interactions for the progeny production of *Prostephanus truncatus* adults treated with spinosad (0.01, 0.1, and 0.5 ppm), DE (150 ppm), or their combinations (error df = 154).

Source	df	*F*	*p*
Temperature	1	2.9	0.09
Treatment	6	252.0	<0.01
Temperature × treatment	6	2.6	0.02

**Table 8:** Mean numbers ± standard errors (SEs) of *Prostephanus truncatus* adults following the exposure of parents to maize treated with spinosad (0.01, 0.1, and 0.5 ppm), DE (150 ppm), or their combinations at 25 and 30 °C. Within each row, asterisks indicate significant differences (df = 14; two-tailed *t*-test at *p* = 0.05). Within each temperature, means followed by the same uppercase letter are not significantly different (df = 7, 95; Tukey HSD test at *p* = 0.05). For entries marked with dashes, no analysis was conducted.

	Temperature	25 °C	30 °C	*t*	*p*
Treatment					
Control		26.8 ± 0.8 A	38.6 ± 1.7 A *	6.7	<0.01
Spinosad 0.01		1.3 ± 1.3 C	1.8 ± 1.8 C	0.1	0.94
Spinosad 0.1		0.0 ± 0.0 C	0.0 ± 0.0 C	-	-
Spinosad 0.5		0.0 ± 0.0 C	0.0 ± 0.0 C	-	-
DE		12.4 ± 0.6 B	23.0 ± 1.2 B *	8.7	<0.01
Spinosad 0.01 + DE		0.0 ± 0.0 C	0.0 ± 0.0 C	-	-
Spinosad 0.1 + DE		0.0 ± 0.0 C	0.0 ± 0.0 C	-	-
Spinosad 0.5 + DE		0.0 ± 0.0 C	0.0 ± 0.0 C	-	-
*F*		255.1	271.4		
*p*		<0.01	<0.01		

**Table 9:** ANOVA parameters for main effects and associated interactions for the progeny production of *Prostephanus truncatus* adults treated with *Beauveria bassiana* (2.3 × 10^4^ and 2.3 × 10^6^ conidia/mL), DE (150 ppm), or their combinations (error df = 110).

Source	df	*F*	*p*
Temperature	1	37.0	<0.01
Treatment	4	48.4	<0.01
Temperature × treatment	4	2.5	0.05

**Table 10:** Mean numbers ± standard errors (SEs) of *Prostephanus truncatus* adults following the exposure of parents to maize treated with *Beauveria bassiana* (2.3 × 10^4^ and 2.3 × 10^6^ conidia/mL), DE (150 ppm), or their combinations at 25 and 30 °C. Within each row, asterisks indicate significant differences (df = 22; two-tailed *t*-test at *p* = 0.05). Within each temperature, means followed by the same uppercase letter are not significantly different (df = 5, 71; Tukey HSD test at *p* = 0.05).

	Temperature	25 °C	30 °C	*t*	*p*
Treatment					
Control		26.3 ± 0.7 A	37.2 ± 1.7 A *	6.1	<0.01
*B. bassiana* 2.3 × 10^4^		11.1 ± 0.6 B	11.7 ± 0.8 C	0.4	0.71
*B. bassiana* 2.3 × 10^6^		5.6 ± 0.7 C	8.3 ± 0.6 CD *	2.8	0.01
DE		12.3 ± 0.5 B	22.6 ± 1.1 B *	9.6	<0.01
*B. bassiana* 2.3 × 10^4^ + DE		4.8 ± 0.6 C	8.2 ± 0.9 CD *	3.2	0.01
*B. bassiana* 2.3 × 10^6^ + DE		4.3 ± 0.6 C	6.7 ± 1.1 D	1.8	0.11
*F*		59.3	58.9		
*p*		<0.01	<0.01		

**Table 11:** ANOVA parameters for main effects and associated interactions for the progeny production of *Prostephanus truncatus* adults treated with abamectin (0.001, 0.01, and 0.1 ppm), DE (150 ppm), or their combinations (error df = 154).

Source	df	*F*	*p*
Temperature	1	0.02	0.90
Treatment	6	811.4	<0.01
Temperature × treatment	6	19.4	<0.01

**Table 12:** Mean numbers ± standard errors (SEs) of *Prostephanus truncatus* adults following the exposure of parents to maize treated with abamectin (0.001, 0.01, and 0.1 ppm), DE (150 ppm), or their combinations at 25 and 30 °C. Within each row, asterisks indicate significant differences (df = 22; two-tailed *t*-test at *p* = 0.05). Within each temperature, means followed by the same uppercase letter are not significantly different (df = 7, 95; Tukey HSD test at *p* = 0.05). For entries with dashes, no analysis was conducted.

	Temperature	25 °C	30 °C	*t*	*p*
Treatment					
Control		26.9 ± 0.6 A	36.8 ± 1.6 A *	5.9	<0.01
Abamectin 0.001		2.8 ± 0.5 C	5.9 ± 0.9 C *	3.1	0.01
Abamectin 0.01		0.0 ± 0.0 D	0.0 ± 0.0 D	-	-
Abamectin 0.1		0.0 ± 0.0 D	0.0 ± 0.0 D	-	-
DE		11.7 ± 0.5 B	21.8 ± 0.8 B *	10.2	<0.01
Abamectin 0.001 + DE		0.0 ± 0.0 D	0.0 ± 0.0 D	-	-
Abamectin 0.01 + DE		0.0 ± 0.0 D	0.0 ± 0.0 D	-	-
Abamectin 0.1 + DE		0.0 ± 0.0 D	0.0 ± 0.0 D	-	-
*F*		733.7	741.9		
*p*		<0.01	<0.01		

## Data Availability

The original contributions presented in this study are included in the article. Further inquiries can be directed to the corresponding author.
